# Respiration Monitoring Using Humidity Sensor Based on  Hydrothermally Synthesized Two-Dimensional MoS_2_

**DOI:** 10.3390/nano14221826

**Published:** 2024-11-14

**Authors:** Gwangsik Hong, Mi Eun Kim, Jun Sik Lee, Ja-Yeon Kim, Min-Ki Kwon

**Affiliations:** 1 Department of Photonic Engineering, Chosun University, 30 Chosundae 3-gil, Dong-gu, Gwangju 61452, Republic of Korea; hyung1_2@chosun.kr; 2 Department of Biological Science, Chosun University, 30 Chosundae 3-gil, Dong-gu, Gwangju 61452, Republic of Korea; kimme@chosun.ac.kr (M.E.K.); junsiklee@chosun.ac.kr (J.S.L.); 3 Korea Photonics Technology Institute, Gwangju 61452, Republic of Korea; jykim@kopti.re.kr

**Keywords:** two-dimensional materials, molybdenum disulfide, respiration sensor, hydrothermal, MoS_2_

## Abstract

Breathing is the process of exchanging gases between the human body and the surrounding environment. It plays a vital role in maintaining human health, sustaining life, and supporting various bodily functions. Unfortunately, current methods for monitoring respiration are impractical for medical applications because of their high costs and need for bulky equipment. When measuring changes in moisture during respiration, we observed a slow response time for 2D nanomaterial-based resistance measurement methods used in respiration sensors. Through thermal annealing, the crystal structure of MoS_2_ is transformed from 1T@2H to 2H, allowing the measurement of respiration at more than 30 cycles per minute and enabling analysis of the response. This study highlights the potential of two-dimensional nanomaterials for the development of low-cost and highly sensitive humidity and respiration sensors for various applications.

## 1. Introduction

Respiration refers to the exchange of gases between the human body and the surrounding environment. It is a crucial process that supports human health, sustains life, and facilitates various activities [1,2]. In healthy adults, the typical respiratory rate ranges from 15 to 20 breaths/min, with each breath exchanging approximately 500 mL of air. However, various diseases and health conditions such as heart disease, pneumonia, bronchitis, sleep apnea, and infections such as COVID-19 can cause changes in both respiratory frequency and depth. These changes can serve as important indicators of health status.

There are various methods for measuring respiration, and humidity sensing has emerged as a promising approach for establishing the relationship between human respiration and electrical signals [3,4,5,6,7]. Humidity sensors used in respiration monitoring can be miniaturized and integrated into wearable devices, allowing the detection of changes in gases produced during respiration, such as moisture and CO_2_. Consequently, there is a growing demand for humidity sensors for monitoring respiration. However, conventional humidity sensors based on capacitance measurement methods tend to be large, have low sensitivity, are prone to temperature variations, and have slow response times, which are among their key limitations [2,4].

In recent decades, the development of microelectromechanical systems (MEMS) has led to the creation of compact humidity sensors based on various principles, including electrical capacitance, resistance, resonance, and optical sensing [4,8]. Methods for directly measuring the moisture generated during respiration can be divided into two types based on the measurement technology: electronic humidity sensors using capacitance measurements and those using resistance measurements. Electronic humidity sensors based on capacitance measurements are widely used owing to their advantages, such as temperature stability, low power consumption, excellent linearity, and extended relative humidity measurement range. However, these sensors have several limitations, including complex structures, the need for dedicated capacitance management electronics, complex manufacturing processes, and high sensitivity to small environmental changes [3]. The sensitivity and accuracy of these sensors are limited by the relative scarcity of high-quality, humidity-sensitive materials and the presence of stray charges on the substrate. The materials commonly used for these sensors include porous silicon, ceramics, and organic materials, with polyimide (PI) being widely used as an organic material. However, PI-based humidity sensors typically have a total output range of only 1–2 pF, which requires the use of precise, application-specific integrated circuits (ASICs) to interface with these sensors [9,10]. Materials such as porous silicon and ceramics (e.g., polar aluminum oxide) provide better moisture sensitivity but involve more complex manufacturing processes and are not compatible with traditional CMOS processing. Furthermore, in real-world applications, changes in ambient temperature affect the dielectric properties of moisture-sensitive materials, resulting in changes in capacitance and, thus, inaccurate measurement results. Moreover, when humidity sensors of this type are used in high-humidity environments for extended periods, moisture typically forms on the surface of the sensor. The recovery process is prolonged because the desorption of water molecules is relatively slow, leading to longer recovery times for such sensors.

Resistance-based humidity sensors offer the advantages of small size, low cost, and the possibility of achieving precise measurements [11,12,13]. It is possible to integrate sensor arrays to increase the sensitivity and response. Additionally, flexible or wearable sensors can be manufactured using thin films and flexible substrates, such as polyimide, and flexible contacts, such as graphene. However, resistance-based humidity sensors, particularly those based on oxides and polymers, encounter various issues [3]. These challenges include slow response times that can hinder real-time data acquisition in critical applications. In addition, they often exhibit low reactivity at low humidity levels, making them less effective under dry conditions. Hysteresis, another concern, can lead to inaccuracies in measurements because the sensor output may differ when the humidity is increasing compared with when it is decreasing. Furthermore, these sensors are often sensitive to external environmental factors such as temperature fluctuations and chemical interference, further complicating their reliable operation. Consequently, addressing these limitations is essential for improving the performance and versatility of resistance-based humidity sensors in various industrial and scientific contexts. Therefore, further research is required to identify and address these issues and develop reliable detection materials.

Molybdenum disulfide (MoS_2_), a two-dimensional (2D) material, has great potential as a sensing material due to its high surface-to-volume ratio, large specific surface area, and numerous active sites. These properties make MoS_2_ and other 2D materials advantageous for sensing applications, as they interact effectively with various compounds and molecules [8,14,15,16,17]. 2D transition metal dichalcogenides (TMDs) are generally part of a family of layered semiconductor structures composed of transition metal atoms, such as Mo or W, and chalcogen atoms, such as Se or S. This layered structural configuration results in pronounced anisotropy in the physical and chemical properties, including electrical, mechanical, and thermal characteristics. For instance, MoS_2_ exhibits an intriguing property whereby it attracts polar molecules, such as water molecules, from the atmosphere due to van der Waals forces. This property makes it suitable for applications as a moisture sensor. Despite the presence of covalent bonds between metal (M) and chalcogen (X) atoms within a layer, the weak van der Waals forces holding these layers together render MoS_2_ easily alterable in terms of its electrical state due to this inherent anisotropy. Furthermore, 2D TMD materials have three crystal structures: 1T (trigonal), 2H (hexagonal), and 3R (rhombohedral) [18]. The lattice parameters of 1T MoS_2_ are 5.6 Å and 5.99 Å; those of 2H MoS_2_ are 3.15 Å and 12.36 Å; and those of 3R MoS_2_ are 3.17 Å and 18.38 Å. The electronic properties and adsorption–desorption characteristics of MoS_2_ vary depending on its crystalline state. In the 1T state, it acts as a metal, whereas in the 2H and 3R states it functions as a semiconductor. Monolayer MoS_2_ exhibits a hexagonal or triangular prism-shaped structure.

However, advancements in humidity-detection technologies based on pure MoS_2_ have been limited. These sensors exhibit low reactivity at low humidity levels and longer recovery times, exceeding 30 s, making them slower to respond than traditional electronic humidity sensors. Additional research is required to identify and address the causes of these issues. It is believed that multiple factors contribute to these issues, including changes in the crystal structure or surface state of MoS_2_, doping, variations in the single-layer and multilayer structures (interlayer interactions), and nanostructures. Specifically, this study found that the crystal structure—namely, the 1T and 2H phases formed during synthesis—is associated with sulfur defects and can significantly impact moisture adsorption and desorption. In a recent study, we proposed a respiration sensor based on humidity changes using MoS_2_ and observed that the structure of MoS_2_, specifically whether it is synthesized layer by layer, results in a 1T@2H or 2H structure when using chemical vapor deposition (CVD). When using CVD-synthesized MoS_2_ for resistance-based humidity sensing, we confirmed that it exhibited a fast response time, responding to respiration within 0.4 s [14]. However, variations in thickness and particle size owing to the growth method still pose some challenges, and the impact of other variables cannot be entirely excluded.

In this research, we recrystallized the sequentially synthesized MoS_2_ thin film from a 1T@2H structure to a 2H structure through thermal annealing processes. We observed the effect of structural changes on moisture response without altering particle size or shape. Additionally, we used this material to create a respiration sensor and confirmed its ability to provide rapid respiratory responses. We further determined that it could sufficiently detect changes in body signals by observing respiration changes over time in mice with cancer and healthy mice, making it possible to detect abnormalities in the body.

## 2. Experimental

### 2.1. Synthesis of MoS_2_ by Hydrothermal Method

**Figure 1:**
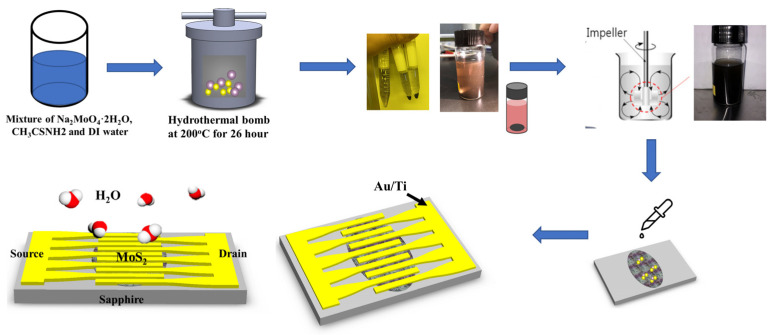
The fabrication process steps for the MaS_2_-based respiration sensor.

MoS_2_ was prepared via hydrothermal synthesis, as shown in Figure 1. For the hydrothermal process of making MoS_2_ samples, 0.15 g of Na_2_MoO_4_·2H_2_O and 0.66 g of CH_3_CSNH_2_ were dissolved in 50 mL of deionized (DI) water and heated for 26 h at 200 °C in an autoclave. Microsized MoS_2_ particles were obtained. The produced MoS_2_ particles were centrifuged, and the remaining solution was discarded. Subsequently, the MoS_2_ particles were re-dispersed in DI water, and the suspension was agitated using an impeller for drop casting. A specified amount of MoS_2_ was deposited onto the sample using drop casting. The deposited MoS_2_ particles were then dried for 10 min at 80 °C.

### 2.2. Fabrication of a Respiration Sensor

Figure 1 shows the humidity and respiration sensors fabricated using MoS_2_. Ti/Au (5 nm/50 nm) electrodes were deposited onto sapphire substrates using e-beam evaporation equipment with a shadow mask to create a cross-fingered, resistive-type sensor, serving as the electron flow channel. The MoS_2_ solution was then applied to the channel area between the two electrodes and allowed to dry.

### 2.3. Measurement of Material Characterization

A 532 nm laser with a power of 0.75 mW was used with a spectrometer (XPER RAM C, Nanobase, Seoul, Republic of Korea) to perform micro-Raman scattering spectroscopy (RSS) on the sample surfaces in ord er to examine the optical properties. The surface morphology of the MoS_2_ synthesized by the hydrothermal technique was examined using an optical microscope (BX-43, Olympus, Tokyo, Japan) and a scanning electron microscope (SNE-4500M, SEC, Suwon, Republic of Korea). X-ray photoelectron spectroscopy (XPS) (K-Alpha+, Thermo Fisher Scientific, Waltham, MA, USA) was used to determine the chemical bonding states of the MoS_2_ thin layers and phase evolution after sulfurization. For the survey, spectra were collected in constant analyzer energy mode with a pass energy of 200 eV using a 400 µm diameter X-ray beam operating at 6 mA and 12 kV. The snapshot acquisition mode (150 eV pass energy) was used to quickly gather narrow regions at a rate of 5 s/region. A flood-gun system, which provides low-energy electrons and low-energy argon ions (20 eV) from a single source, was used to perform charge correction. Thermo Scientific Advantage software V5 was used for data collection and processing. Internal standards of Au, Ag, and Cu were used in conjunction with an automated calibration procedure to perform spectral calibration. The TEM samples were studied using a transmission electron microscope (JEM-2100F HR, JEOL LTD, Tokyo, Japan installed at the National Nanofab Center, Daejeon, Republic of Korea ) at an accelerating voltage of 200 kV. X-ray diffraction (XRD) was used to analyze the crystalline quality of MoS_2_.

### 2.4. Measurement of Characterization of Respiration Sensor

Prior to measuring the respiration sensor response, the current response to humidity changes was examined. This was accomplished using a TR-72WB, a commercially available capacitance-type moisture sensor, to monitor humidity levels, and by measuring the current response of our sensor when the relative humidity was quickly changed from 40% to 60%. The current response was measured using a Parameter Analyzer (4200A-SCS, Keithley, OH, USA). The respiration sensor was mounted on the front of a face mask, and the response was measured according to the rate and extent of water vapor changes during respiration. We verified the sensor’s response to moisture in this range because respiration-induced variations in moisture content often occur between 40% and 70% relative humidity. The sensor was secured to the front of the face mask, and the current response was examined by connecting it to a parameter analyzer.

### 2.5. Mice

Male C57BL/6 mice (6 weeks old) were purchased from Orient Bio, Inc. (Sungnam, Republic of Korea) All mice were maintained in a pathogen-free environment for one week at a controlled temperature with a 12 h light/dark cycle.

## 3. Results

### 3.1. Crystal Structures of MoS_2_

**Figure 2:**
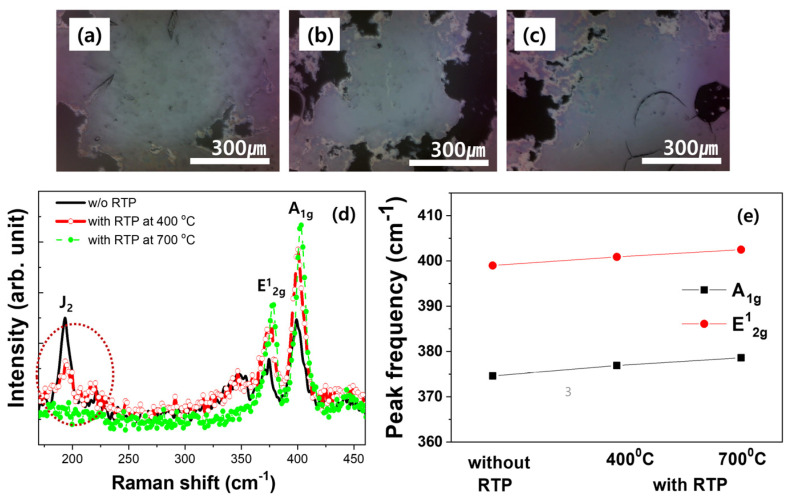
Optical microscope images at different annealing temperatures: (**a**) before annealing, (**b**) at 400 °C, (**c**) at 700 °C; (**d**) Raman spectrums of MOS_2_ without and with annealing (400 and 700 °C) and; (**e**) peak frequency difference between A_1g_ and E^1^_2g_ peaks.

For MoS_2_ samples synthesized via hydrothermal synthesis, we employed optical image analysis and Raman spectroscopy, as shown in Figure 2. The rapid thermal annealing (RTP) was performed at 400 °C and 700 °C in a nitrogen (N_2_) gas atmosphere, an inert gas that does not react with MoS_2_ thin films. As the annealing temperature increased, recrystallization of the thin film was induced. Upon examining the optical images, we observed no significant differences between the images of MoS_2_ before and after thermal annealing, as illustrated in Figure 2a–c. However, Raman spectroscopy revealed a slight decrease in the intensity of the 1T@2H structure in the 200 cm^−1^ region after thermal annealing at 400 °C and 700 °C. As thermal annealing progressed, the 1T structure completely disappeared from the 200 cm^−1^ region (J_2_ mode), transforming into a 2H structure similar to MoS_2_ grown by chemical vapor deposition (CVD).

The following statements explain how the anion exchange and reduction reactions in the hydrothermal approach led to the formation of MoS_2_.

CH_3_CSNH_2_ + 4H_2_O → H_2_S + NH_3_ + 2CO_2_ + 4H_2_ (1)

H_2_S + OH^−^ → S^2−^ (2)

MoO_3_ + 3S^2−^ → MoS_3_ (intermediate) → (1T/2H) MoS_2_ (3)

As a result of the hydrolysis process, CH_3_CSNH_2_ first breaks down to form S^2−^ anions. The S^2−^ anions then react with MoO_3_ to generate MoS_3_ (intermediate). The metastable mixed 1T and 2H phases may be attributed to possible intercalation of water molecules at higher temperatures, resulting in a change in the interlayer spacing along the c-axis. Consequently, the final forms are the 1T and 2H phases of MoS_2_ derived from MoS_3_.

When thermal annealing is performed, S defects are significantly reduced by the rearrangement of atoms, and the d-spacing along the c-axis increases. This indicates that the 1T structure can be changed into the 2H structure, which is a stable phase.

Furthermore, with increasing temperature, both the intensity and sharpness of the E^1^_2g_ and A_1g_ peaks in the Raman spectra increase, confirming an enhancement in the crystallinity of the MoS_2_ thin film. Additionally, there is a slight rightward shift in the peak positions of E^1^_2g_ and A_1g_, indicating a minor atomic structure adjustment in both the in-plane and out-of-plane directions.

We conducted X-ray diffraction (XRD) and X-ray photoelectron spectroscopy (XPS) analyses, in addition to Raman spectroscopy, to further examine the thin-film properties before and after thermal annealing. Upon initial inspection of the XRD patterns, a noticeable difference in the (002) crystal plane was observed before and after thermal annealing. In the specimen without thermal annealing, the XRD peak intensity was very low, indicating poor crystallinity. With thermal annealing, the intensity of the (002) peak significantly increased, indicating crystallization. The presence of the (002) peak indicates the formation of a 2H structure, which is consistent with our previous Raman measurements.

**Figure 3:**
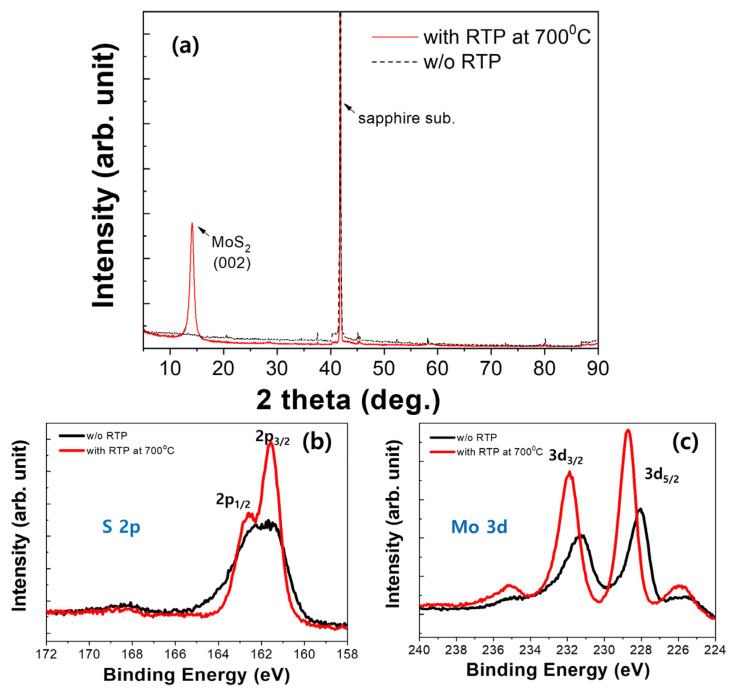
(**a**) XRD spectra and S 2p and Mo 3d XPS spectra of MoS_2_ (**b**) with and (**c**) without thermal annealing.

As shown in Figure 3b,c, the sample initially synthesized hydrothermally without thermal annealing shows that the 3d_3/2_ and Mo 3d_5/2_ peaks are at 231.4 and 228.4 eV, respectively. These values are lower than the known Mo^4+^ peak values for 2H MoS_2_. Hydrothermal synthesis produces more S defects than in 2H crystalline MoS_2_. Additionally, the S 2s peak of the as-grown MoS_2_ was at a lower binding energy than the 3d_3/2_ and 3d_5/2_ peaks. Furthermore, the S 2p peak also indicated that the binding energy of the as-grown hydrothermally synthesized MoS_2_ was lower than that of the thermally treated MoS_2_. It is evident that, in the case of the as-grown hydrothermally synthesized MoS_2_ before thermal annealing, the peak’s full width at half maximum (FWHM) was broad. This indirectly indicates the presence of a thin film with many defects, such as S defects, that prevent the proper bonding of Mo and S ions, thereby lowering the bonding energy. After thermal annealing, the Mo 3d_3/2_ and Mo 3d_5/2_ peaks shift to higher energies, and the S peak exhibits a reduced FWHM and increased intensity. These results are consistent with the Raman and XRD measurements, which show a reduction in S defects and the generation of the 2H phase. Based on the area ratio before and after thermal annealing, the purity of the 1T and 2H phases was calculated [19]. It was observed that the 1T phase decreased from 94.5% to 20.6%, while the 2H phase increased significantly from 5.5% to 79.3% after thermal annealing. (Supplement Figure S1).

**Figure 4:**
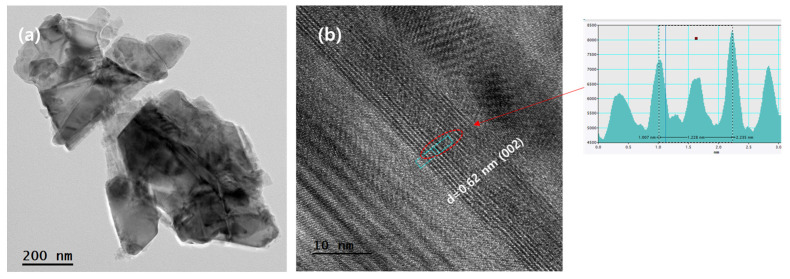
TEM images of MoS_2_ annealed at 700 °C: (**a**) low magnification and (**b**) high-resolution TEM image.

Figure 4 shows the TEM images of MoS_2_ annealed at 700 °C. The low-magnification images show that the size of the particles is approximately 400–600 nm, and they are composed of a multilayered structure. The corresponding high-resolution image is shown in Figure 4b. A lattice spacing of 0.62 nm can be observed; this was assigned to the (002) crystal plane of 2H-MoS_2_. This clearly confirms the presence of 2H-MoS_2_ after annealing at 700 °C.

### 3.2. Resistive-Type Respiration Sensor with MoS_2_

**Figure 5:**
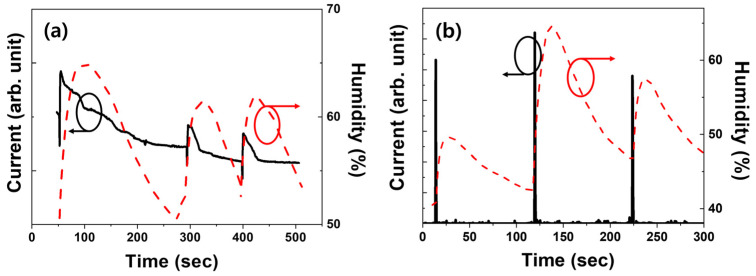
Current response to humidity change for resistive-type, MoS_2_-based respiration sensors (**a**) without and (**b**) with thermal annealing.

First, we compared the performance of MoS_2_ thin films produced by hydrothermal methods using cross-fingered, resistive humidity sensors. For comparison, we measured the humidity in the range of approximately 40–70% using a commercial capacitive sensor (TR-72WB). Rapid humidity fluctuations are difficult to measure owing to the limitations of conventional capacitive-type sensors. Therefore, measurements were conducted while maintaining the breathing cycle at over 100 s through humidity changes induced by breathing ranging between 40% and 70%. Although the relative humidity near the nasal cavity can reach 100%, this study focused on the rapid adsorption and desorption of moisture during breathing. Therefore, the initial experiments were conducted under the assumption that the actual variation would fall within the 40–70% range. The current for both sensors increased as humidity increased, as shown in Figure 5a. Adsorbed water molecules can dissociate on the MoS_2_ surface at ambient temperatures, forming hydroxyl groups (OH^−^) that are chemically adsorbed on MoS_2_ and function as electron donors. This enhances the conductivity of the n-type MoS_2_ semiconductor and boosts the current. The sensor using the as-grown hydrothermally synthesized MoS_2_ exhibited a quick response to water vapor absorption, but the recovery period was long, as shown in Figure 5a. Al- though the manufactured sensor had a faster recovery compared with commercially available sensors, the recovery time was still too long to detect rapid respiration effectively.

Similar outcomes have been previously reported [13]. To use MoS_2_ as a respiratory sensor, it is crucial to identify whether there is an issue with the MoS_2_ film’s ability to adsorb moisture or whether other external factors are at work. When the humidity was increased from 40% to 55%, the current response of the humidity sensor made of heat-treated hydrothermally grown MoS_2_ increased. As shown in Figure 5b, the sensor fabricated from the heat-treated MoS_2_ exhibited a significantly faster response and recovery speed than that made from as-grown MoS_2_. This indicates that the manufactured sensor is extremely sensitive to variations in humidity. However, these findings differ from those of earlier investigations of sensors with slow recovery times. Previous studies have reported a significant improvement in the reaction rate of samples synthesized using the CVD method compared with those produced by the hydrothermal method [13]. Differences in the crystal structures were also suggested to have played a role in determining the reaction rate. However, owing to the variations in size, shape, and thickness, it was challenging to clearly identify the causes of the differences in the reaction rates. In this study, we maintained the same synthesis method and induced changes in crystalline quality through thermal annealing without altering the morphology of the sample. Therefore, we concluded that the 2H crystal structure significantly influences moisture adsorption and desorption. Bobbitt et al. reported that sulfur defects bind strongly to water vapor, more than doubling the binding strength. Hence, such strong binding is expected to affect desorption [18]. Therefore, it can be stated that changes in the crystal structure, specifically the transition from a 1T@2H structure to a 2H structure, are essential for creating highly sensitive respiratory sensors for measuring breath responses. The current response to a 30–80% change in relative humidity for this specimen is shown in Supplementary Figure S2.

**Figure 6:**
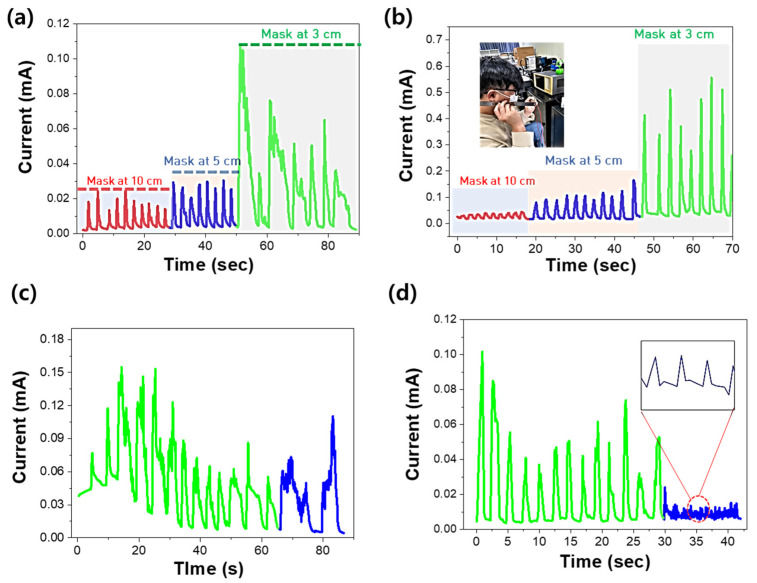
Current response of MoS_2_-based sensors depending on distance between nose and sensor: (**a**) without thermal annealing and (**b**) with thermal annealing; and current response to normal and fast breathing with MoS_2_: (**c**) without thermal annealing and (**d**) with thermal annealing.

Sensors with MoS_2_ were periodically tested at distances of 3, 5, and 10 cm to track their reactivity based on the distance between sensor and nose as shown in Figure 6. As the sensor–nose distance decreased, the reactivity increased, mainly because of the significant changes in moisture levels at shorter distances. However, the sensors without thermal annealing exhibited reduced sensitivity at a distance of 3 cm, possibly due to overlapping signals as shown in Figure 6a. Sensors utilizing MoS_2_ with thermal annealing demonstrated rapid response capability owing to their high adsorption and desorption rates as shown in Figure 6b. This enabled the detection of both rapid and slow breathing patterns. Furthermore, the heat-treated specimens provided precise detection.

**Figure 7:**
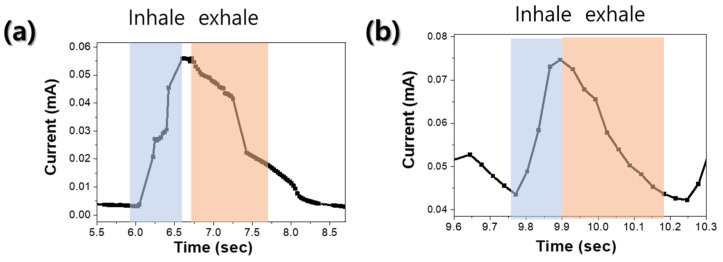
Current response of single breathing with sensor using MoS_2_ (**a**) without and (**b**) with thermal annealing.

Figure 7 shows the current response of single breathing with respiration sensor using MoS_2_ (a) without and (b) with thermal annealing. When the reaction and recovery times were measured as explained above, the unannealed respiration sensor showed a slow response and recovery, with a response time of 0.7 s and a recovery time of 1.5 s. Therefore, it would be difficult to use this sensor as a breathing sensor. The respiratory sensor that was finally heat-treated at 700 °C showed the fastest reaction and recovery times of 0.12 and 0.21 s, respectively. However, based on the characteristics shown in the graph, the recovery speed appears to be slower than the reaction speed. To improve the relatively slow recovery speed, it is deemed necessary to conduct research aimed at enhancing the crystal quality of MoS_2_ thin films, such as reducing S vacancies, which affect H_2_O desorption [20].

### 3.3. Tracking and Managing Mice with Lung Cancer and Healthy Mice

**Figure 8:**
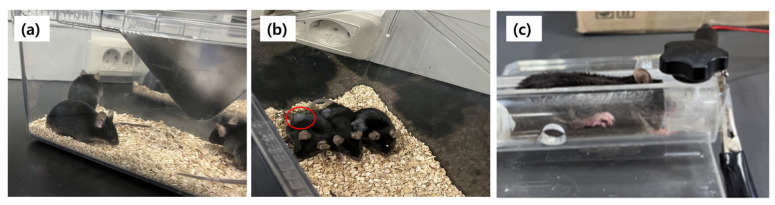
(**a**) Mice without cancer cell injection; (**b**) mice injected with cancer cells; and (**c**) mouse set up for measurement of respiration response. The red dot ring in (**b**) shows the location and appearance of cancer cells.

We compared the respiratory responses of mice injected with cancer cells with those of healthy mice without cancer cell injections. As shown in Figure 8, cancer cells were injected into the colon and metastasized to the lungs. Therefore, mice injected with cancer cells exhibited noticeable convex protrusions in the areas of the colon and lungs, which were not observed in mice without cancer cell injections. The sensor measurement method involved immobilizing the mice in a holder, exposing only their noses, and positioning the sensor nearby for measurement.

**Figure 9:**
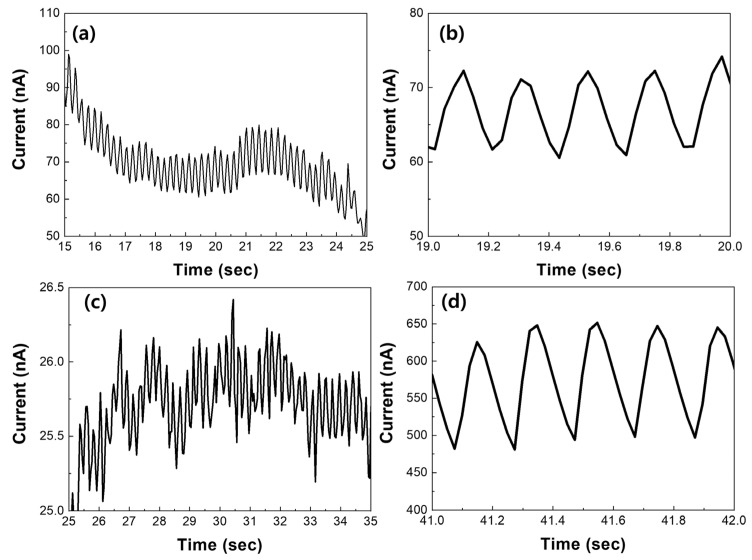
Respiratory responses of healthy mice (**a**,**b**) and cancer-bearing mice (**c**,**d**): (**a**) week 3, (**b**) zoom in data of (**a**), week 3, (**c**) week 0, (**d**) week 3.

Figure 9 shows the current changes for healthy mice and mice with cancer. As seen in Supplementary Figures S3 and S4, the initial current values in response to respiratory changes (week 0) were similar, measuring below 1 nA. As the mice grew and the tumors enlarged, the current change in response to respiratory variations increased to 150 nA, more than ten times greater than the 12 nA change observed in healthy mice as shwon in Figure 9b,d. Additionally, it was noted that the breathing cycle became shorter, indicating that the mice’s breathing became irregular and strained. This substantial increase in current values could be attributed not only to the natural growth of the mice but also to the release of volatile organic compounds from the cancer cells. These results suggest that the developed sensor has potential for use as a diagnostic tool for lung cancer. Further precise research is needed to better understand the changes induced by cancer and the effects of volatile organic compounds.

## 4. Conclusions

In this study, we found that the low responsivity of the MoS_2_-based respiration sensor can be attributed to the 1T@2H crystalline structure and sulfur defects. The response and recovery speeds significantly improved when high-quality 2H crystal structures were formed via thermal annealing, making the sensors more sensitive to respiratory reactions. Additionally, experiments on mice with cancer confirmed that these sensors can detect abnormal signals in the body. These findings could serve as a foundation for technologies that could significantly improve human health by monitoring and preventing major diseases or accidents through the detection of abnormal breathing patterns.

## 5. Patents

Part of this study is the subject of a pending Korean patent application (10-2022-0064528).

## Data Availability

Data are contained within the article.

## Supplementary Materials

The following supporting information can be downloaded at https://www.mdpi.com/article/10.3390/nano14221826/s1, Figure S1: XPS spectra of MoS_2_ (a) before and (b) after thermal annealing; Figure S2 Current response of MoS_2_ based humidity sensor as function of humidity change, Figure S3 Current response of healthy mice without cancer cell injections after (a) 0 weeks, (b) 1 week, (c) 2 weeks, (d) 3 weeks, Figure S4 Current response of mice with cancer cell injections after (a) 0 weeks, (b) 1 week, (c) 2 weeks, (d) 3 weeks.
