# Supplementary figures and images for: Respiration Monitoring Using Humidity Sensor Based on Hydrothermally Synthesized Two-Dimensional MoS2
Source: Nanomaterials (Basel). 2024 Nov 14;14(22):1826. doi: 10.3390/nano14221826 (PMC11597190; doi:10.3390/nano14221826)

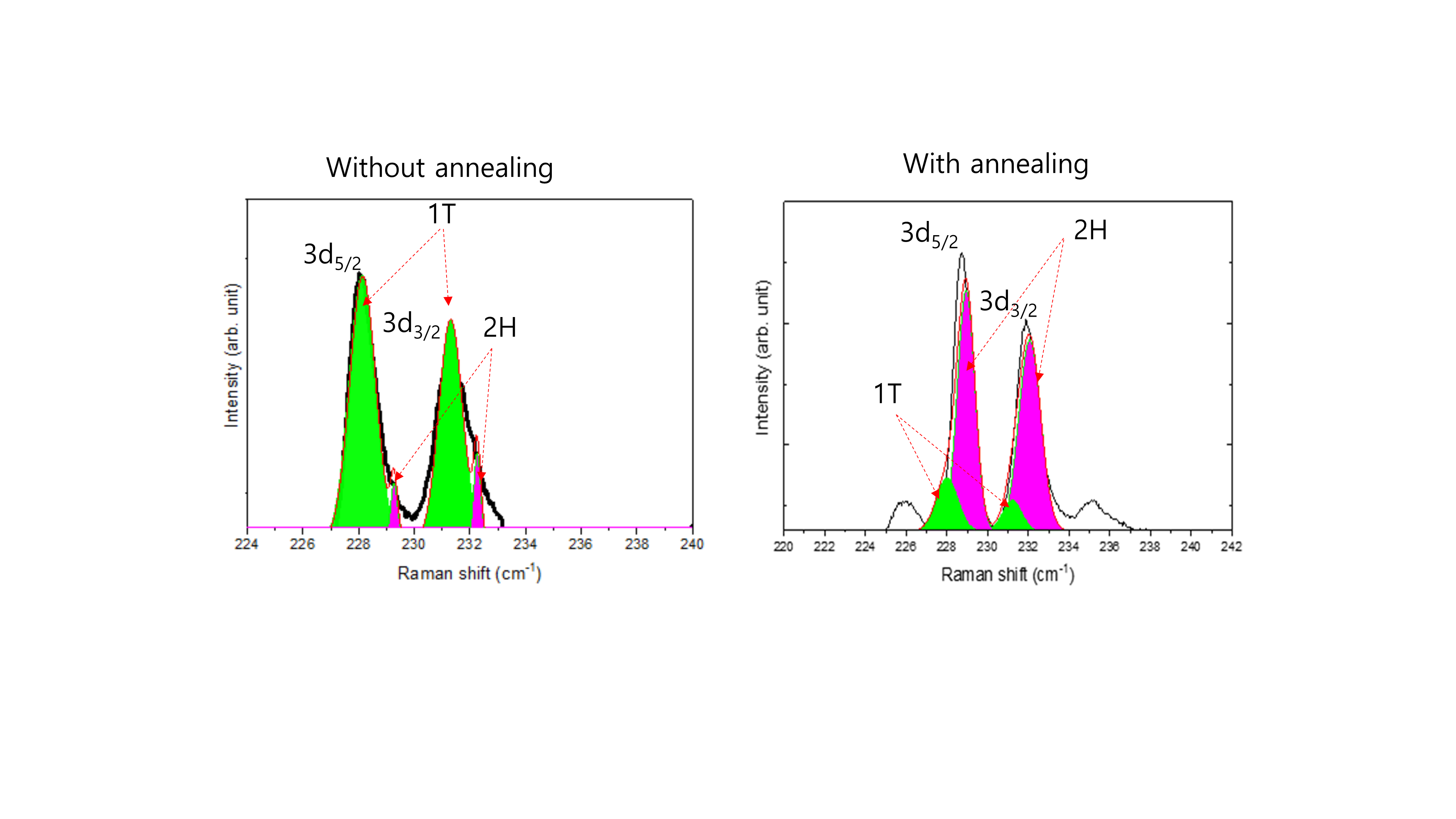

Supplement: Supplementary file 1 [file nanomaterials-14-01826-s001.zip › supplementray fig S1.tif]

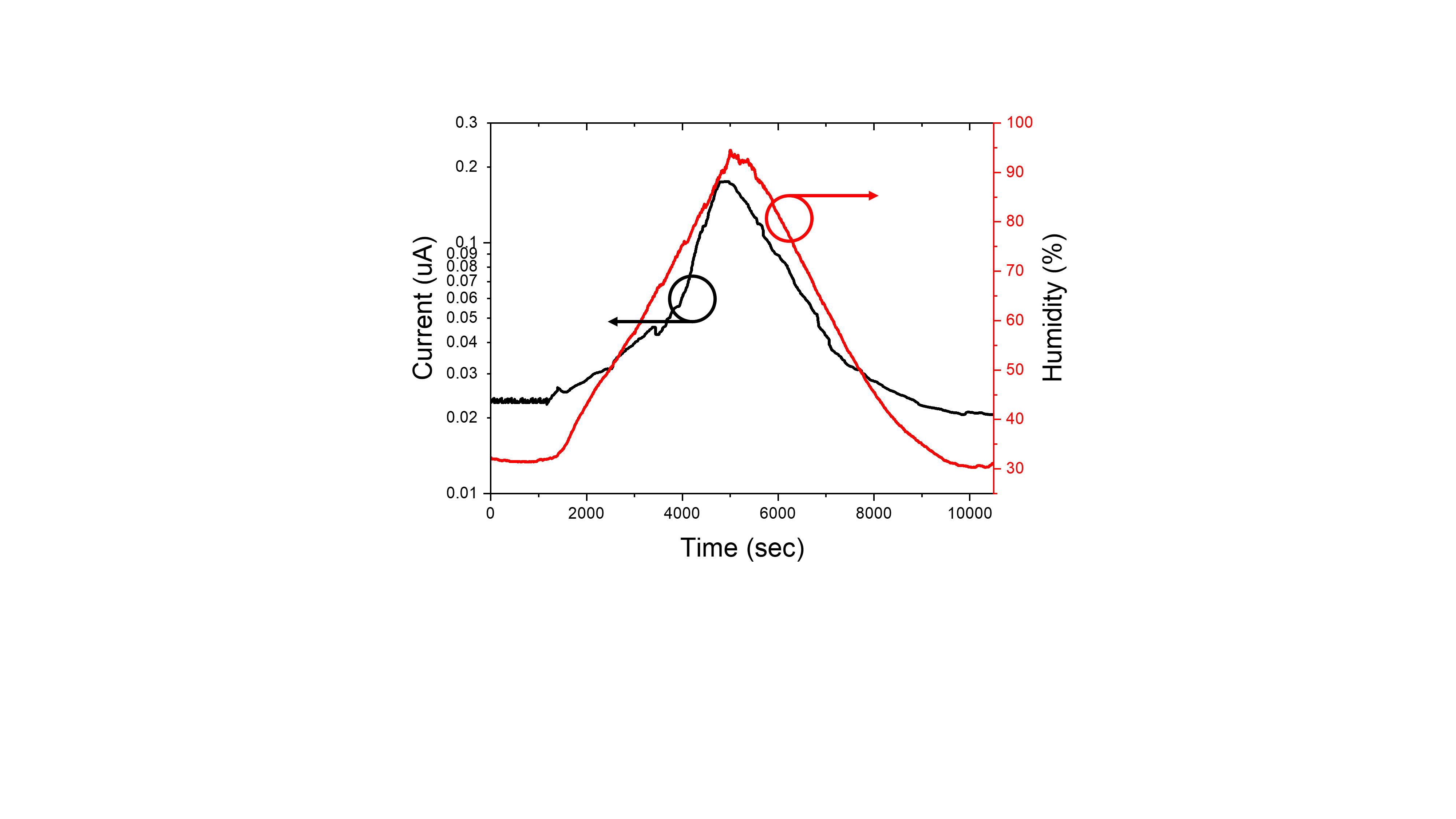

Supplement: Supplementary file 1 [file nanomaterials-14-01826-s001.zip › supplementray fig S2.tif]

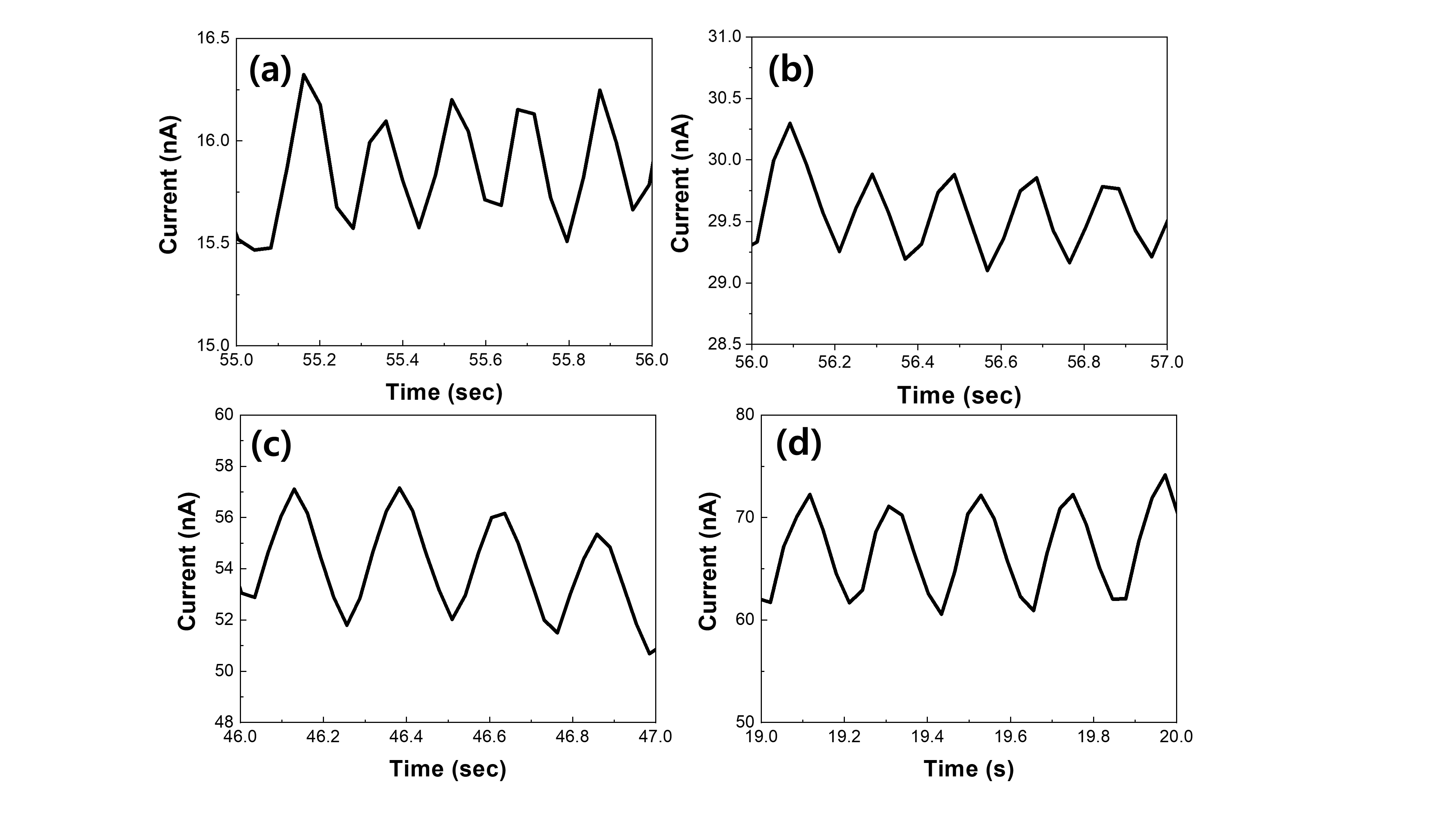

Supplement: Supplementary file 1 [file nanomaterials-14-01826-s001.zip › supplementray fig S3.tif]

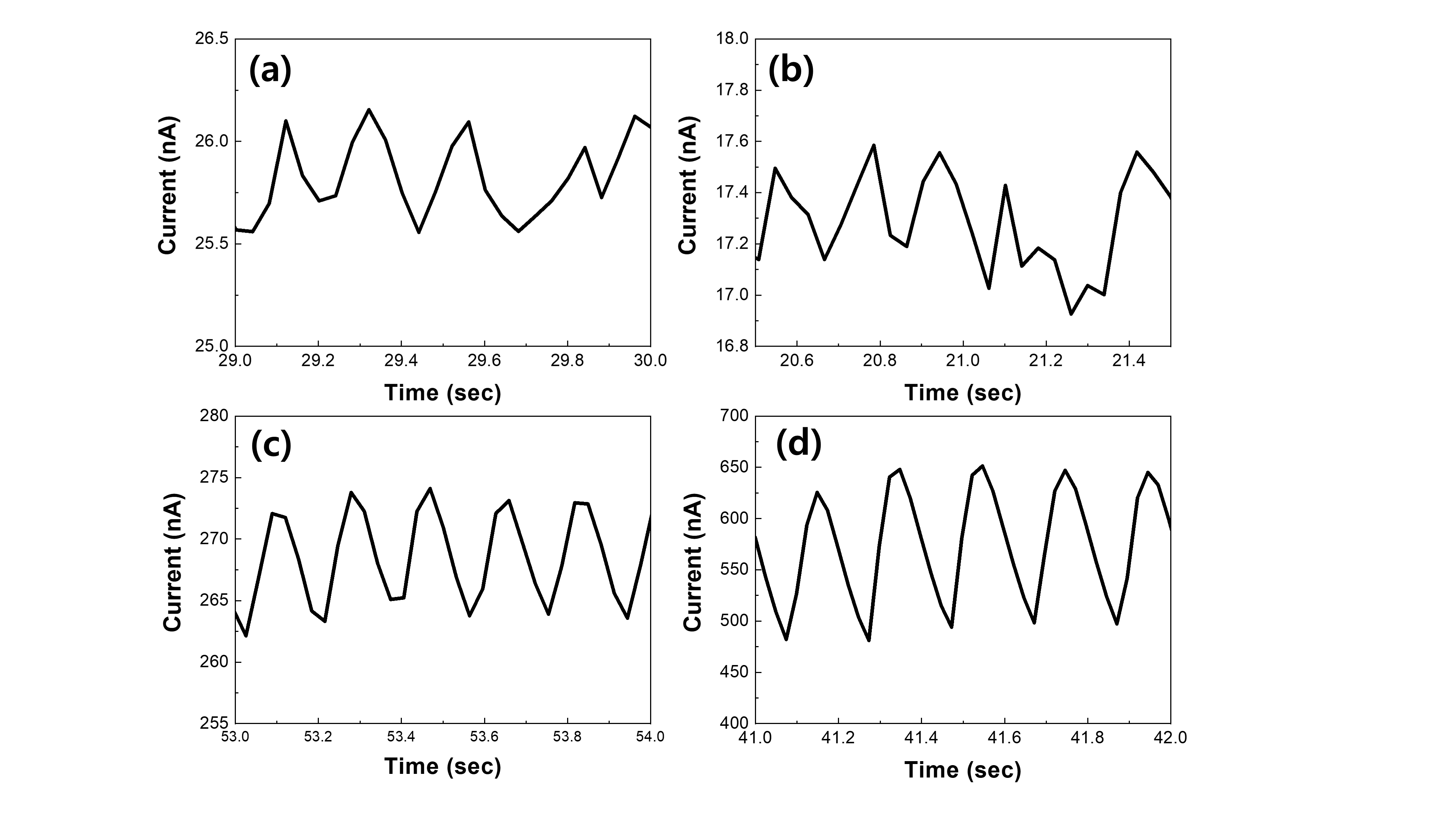

Supplement: Supplementary file 1 [file nanomaterials-14-01826-s001.zip › supplementray fig S4.tif]
